# DNA Damage and Repair in Epithelium after Allogeneic Hematopoietic Stem Cell Transplantation

**DOI:** 10.3390/ijms131215813

**Published:** 2012-11-27

**Authors:** Maria Themeli, Alexandros Spyridonidis

**Affiliations:** Hematology Division, BMT Unit, University Hospital of Patras, Rio 26500, Greece

**Keywords:** bone marrow transplantation, mismatch repair, microsatellite instability, graft-*versus*-host reaction

## Abstract

Allogeneic hematopoietic stem cell transplantation (allo-HSCT) in humans, following hematoablative treatment, results in biological chimeras. In this case, the transplanted hematopoietic, immune cells and their derivatives can be considered the donor genotype, while the other tissues are the recipient genotype. The first sequel, which has been recognized in the development of chimerical organisms after allo-HSCT, is the graft versus host (GvH) reaction, in which the new developed immune cells from the graft recognize the host’s epithelial cells as foreign and mount an inflammatory response to kill them. There is now accumulating evidence that this chronic inflammatory tissue stress may contribute to clinical consequences in the transplant recipient. It has been recently reported that host epithelial tissue acquire genomic alterations and display a mutator phenotype that may be linked to the occurrence of a GvH reaction. The current review discusses existing data on this recently discovered phenomenon and focuses on the possible pathogenesis, clinical significance and therapeutic implications.

## 1. Introduction

Over the last 30 years, allo-HSCT has become a standard treatment for many hematological malignancies and is now increasingly used also as immunotherapy for the treatment of solid tumors [1,2]. During allo-HSCT, the recipient receives a preparative conditioning regimen (e.g., chemotherapy, radiotherapy) to destroy his hematopoiesis and immune system. This practice is followed by the administration of Hematopoietic Stem Cells (HSC) harvested from the donor. The donor’s HSC engraft, proliferate and finally reconstitute hematopoiesis in the recipient [3,4]. The result is the creation of a biological chimera, a term used to describe the presence of tissues of different genetic origin in the same organism; the hematopoietic cells derive from the donor, while the other tissues (e.g., epithelium) are genetically derived from the patient-recipient. This unphysiological formation of biological chimeras is not free of consequences. After allo-HSCT, epithelial tissues become injured through the preparative regimen and are then potentially attacked by allo-reactive T cells. The net effect of these allo-antigeneic reactions is tissue stress and apoptosis, which we recognize clinically as Graft-*versus*-host Disease (GvHD) [5].

DNA is a reactive molecule and is constantly attacked and modified by external and internal agents. A mammalian DNA molecule can undergo about 100,000 modifications per day and replicates with an error rate of one error per 10^10^ nucleotides [6]. Genome integrity is a basic element of cellular homeostasis. To maintain genome integrity and ensure its stable inheritance during replication, cells are equipped with several mechanisms, including DNA repair, cell-cycle checkpoints and programmed cell death [7]. The term genomic instability (GI) describes the failure to transmit an accurate copy of the entire genome from one cell to its two daughter cells. The existence of genomic instability is a sign of the failure of the protection mechanisms and has been correlated with cancer and cancerous transformation [8].

Microsatellites are short tandem repeat sequences (repeat units range from 1–6 bp in length) dispersed throughout the genome. There are more than 1 million microsatellite loci in the human genome, which comprises approximately 3% of the genome. Microsatellite sequences are among the most variable types of DNA sequence in the genome. Their polymorphism derives mainly from variability in length, rather than in primary sequence. Though highly polymorphic, as their length varies in a population, they are inherited stably and are unique to each individual, which means that the length of a microsatellite is the same in all the cells of the same person [9]. For this reason, the detection of different MS polymorphisms with PCR-based assays can be used for linkage mapping, paternity testing and forensic purposes, as well as for chimerism quantification after allo-HSCT [9,10]. The term microsatellite instability (MSI) describes alterations in the length of a microsatellite locus detected by PCR amplification of an individual’s DNA [7]. Such length alterations could refer to insertion or deletions of repetitive units, which may be the result of polymerase slippage during DNA replication [11]. The repetitive nature of microsatellites makes them especially prone to such damage [11,12]. According to several studies, there are indications that the DNA structure of these repetitive sequences is not in the normal B-conformation *in vivo* and, therefore, binds poorly to DNA repair enzymes [13], which makes it susceptible to DNA damage. Therefore, the detection of MSI indicates a general GI, where mutations in DNA can be accumulated and which may be related to carcinogenesis [9,14]. MSI was first detected and described in individuals with a type of hereditary colon cancer (HNPCC, hereditary non-polyposis colorectal carcinoma), which is caused by mutation of the DNA-mismatch-repair gene *MSH2*[14]. In addition to HNPCC, MSI has also been reported in several sporadic cancers (colorectal, bladder, skin, lung and ovarian) [14–17]. Recently, MSI has also been observed in chronic inflammatory diseases, such as ulcerative colitis and rheumatoid arthritis [18,19]. The exact mechanisms through which chronic inflammation may lead to these genomic alterations are the subject of continuing debate. The biologic significance of genomic instability in chronic inflammation settings is yet unknown. In ulcerative colitis, MSI has been associated with increased cancer risk [20], while there is no such indication in rheumatoid arthritis [19].

Faber *et al.*[21] hypothesized that the chronic tissue stress resulting after allo-HCT may cause genomic alterations in host epithelium. The donor-derived lymphocytes may interact with and damage recipient epithelial cells at the molecular level in these chimeric individuals. Examination of tissues isolated from patients treated with allo-HCT revealed frequent genomic alterations, which were detected as microsattelite instability (MSI). These genomic alterations were found only in allogeneic transplanted patients, but not after autologous HSCT or intensive chemotherapy, and therefore, they suggested that factors implicated in the alloreactive microenvironment after allo-HSCT are substantially involved in the mutation process. Further analyses performed by our group [22] in patients who underwent allo-HSCT also confirmed the presence of frequent MSI in the non-neoplastic tissues of allogeneic transplanted patients. This was most frequently found in older patients, in males transplanted with cells from female donors and in patients that experienced an extreme GvH reaction. In an *in vitro* mutation analysis system, we found that allostimulated mixed lymphocyte cultures may induce MSI in co-cultured epithelial cells [22]. These results were independently confirmed by subsequent studies from other groups, which reported that the detection of MSI [23] and chromosomal instability [24] in the epithelial tissues of patients after allo-HSCT was correlated to the presence of GvHD inflammation.

## 2. What Does the MSI Found in Non-Neoplastic Tissues after Transplantation Point Out?

**Figure 1:**
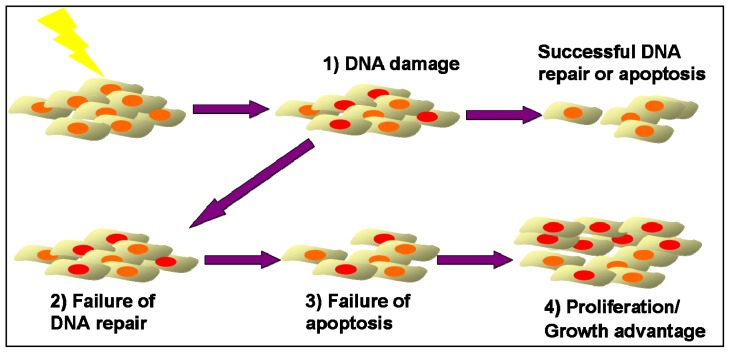
Emergence of genomic instable clones after allogeneic HSCT. (**1**) A cell population is constantly exposed to an allogeneic factor that causes DNA damage; (**2**) Some cells fail to repair DNA damage through DNA repair mechanisms; (**3**) Failure of apoptosis of damaged cells through DNA-damage checkpoints activation; (**4**) Proliferation of damaged cells, and perhaps acquisition, of growth advantage so as to create a detectable clonal population.

Replication errors during DNA synthesis occur with a defined probability in all cells and may result in a change in the length of microsatellite loci. The mismatch repair (MMR) system recognizes and corrects these genomic alterations [6]. Whenever a DNA error cannot be repaired, apoptosis pathways are activated [7]. Thus, cells with microsatellite alterations, which escaped repair, will not replicate and, therefore, in molecular studies, should not become apparent among the large excess of the surrounding normal cells [25]. However, if deficient DNA repair is coupled with a failure to elicit an apoptotic response, this association may result in a growth advantage sufficient to generate a detectable clonal population of cells that share genetic alterations. Therefore, the detection of MSI in clinical samples indicates the presence of a cell population, which (i) was exposed to a factor that caused MSI, (ii) failed to repair the genetic damage through normal DNA repair mechanisms, (iii) were not eliminated by apoptosis via activation of DNA-damage checkpoints and finally (iv) multiplied, and perhaps acquired, a growth advantage so as to create a detectable clonal population (Figure 1).

## 3. Possible Mechanisms Explaining Genomic Instability after Allo-HCT

### 3.1. Factors Inducing GI in the Allotransplanted Recipients

As mentioned before, MSI in non-neoplastic epithelial tissues has been found only in patients treated with myeloablative or reduced-intensity chemotherapy and allogeneic HSCT, but not in patients after myelosuppressive chemotherapy or myeloablative chemotherapy combined with autologous HSCT. In addition, MSI was found many years after HSCT [21]. It is very likely that the alloantigeneic GvH reactions and the following tissue stress could be the driving force in producing detectable MSI in the allografted patients. Several hypotheses could be made on which of the elements of this inflamed environment could be responsible for causing MSI and by which mechanism. During GvHD, donor activated lymphocytes and macrophages are recruited in the patient’s tissues [5,26]. These activated cells produce mediator molecules, such as cytokines (IFN-γ, TNF-α, IL-1 *etc*.), and reactive oxygen species (ROS), such as H_2_O_2_, OH·, O_2_·. These factors have been shown to cause DNA damage in hematopoietic cells of mice with GvHD [27] and in various other experimental systems [28].

ROS can cause base pair mutations, deletions and insertions among other DNA structural alterations through different mechanisms [28]. Oxidants have been shown to induce mutations directly by chemical modification of DNA bases or conformational change of DNA that diminishes the accuracy of DNA polymerases [28]. *In vitro* experiments demonstrated that H_2_O_2_ can cause induction of MSI, not only in MMR-deficient, but also at higher concentrations in MMR-proficient human colorectal cancer cell lines [29]. Thus, even with an intact MMR system, free radicals produced in inflamed tissues could cause such direct DNA damage, which overwhelms the capacity of repair pathways. Oxidants may also facilitate the accumulation of mutations and the creation of MSI indirectly by oxidative damage of DNA repair proteins [30] or through DNA methylation and silencing of DNA repair genes [31]. Besides the effect of oxidants in the DNA repair machinery, ROS may also alter the DNA-damage checkpoints, such as p53, facilitating in this way the survival of cells with genomic alterations [32].

Inflammatory cytokines may also induce DNA damage in epithelial cells [33,34]. A nitric oxide (NO) pathway is mainly involved in the cytokine-induced DNA damage. Cytokines, such as TNF-α and IFN-γ, which are released in increased amounts in GvHD areas, may cause activation of nuclear factor κB (NFκB), which induces the expression of iNOS (inducible NO synthase) in the epithelial cells. NO produced by cytokine activated cells can be auto-oxidized, leading to the formation of reactive radicals, called reactive nitrogen species (RNS), which, like ROS, are mutagenic agents with the potential to chemically modify DNA bases by deamination, nitration or oxidation. In addition, NO may affect DNA repair processes by inhibiting DNA repair enzymes through sulfhydryl-nitrosylation of cystein residues of their DNA binding sites [28,33]. Therefore, NO might be another key player in the induction of MSI after allo-HCT. NO may indeed be the link between chronic alloantigeneic stimulation, relaxation of DNA repair mechanisms and GvHD induced genomic instability. NO has been shown to be an important mediator in GvHD pathology [26]. Increased levels of circulating NO characterize GvHD and iNOS inhibition leads to decreased GvHD severity in animal models [35,36]. In addition, plasma levels of NO have been associated with GvHD severity in humans [37].

Taken together, oxidative stress, like the one produced in the biological chimera due to the interaction between donor T cells and host epithelium, may lead to accumulation of genomic alterations in the recipient cells, either by overwhelming the capacity for DNA repair or by directly inactivating DNA repair pathways.

### 3.2. Failure of DNA Repair

**Figure 2:**
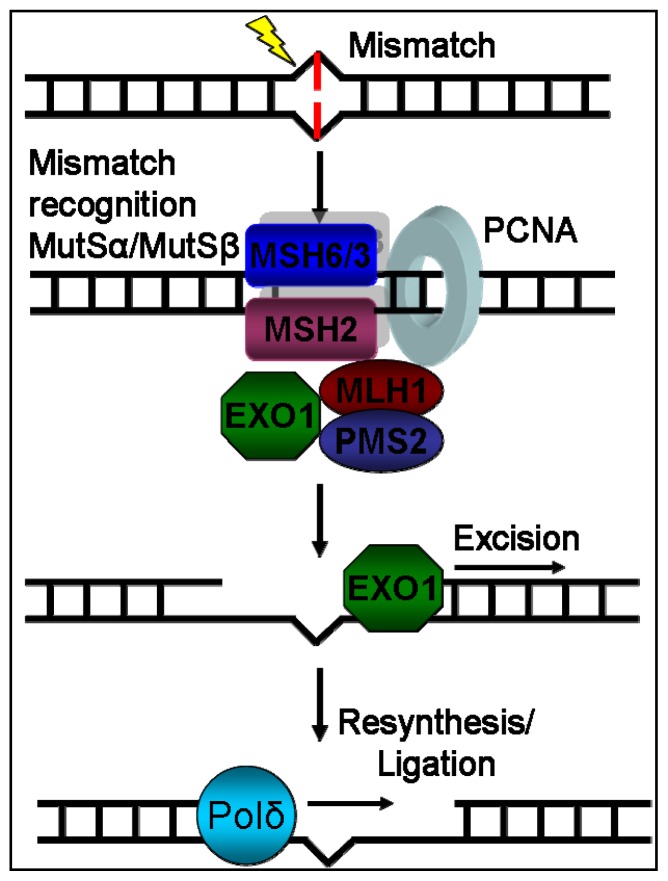
Representation of MMR system’s mechanism of action. DNA damage recognition is mediated through the binding of MutSa (MSH2-MSH6) or MutSβ (MSH2-MSH3) complex. Consecutively, the MutLa (MLH1-PMS2) complex binds to the machinery. After the binding of both complexes, the machinery moves across the DNA by using ATP molecule hydrolysis and recruits EXO1, which excises the damaged DNA part. Finally, the correct new DNA is reconstructed by Polδ.

The presence of MSI in non-neoplastic tissues after allo-HSCT means that a replication error occurred and escaped from the DNA repair mechanisms. As mentioned before, oxidative stress may influence the function of the DNA repair machinery. The normal function of the MMR system has been identified as crucial to microsatellite stability [6,11]. It consists of several proteins (MSH2, MSH3, MSH6, MLH1 and PMS2) with different functions that recognize base-base mismatches or insertion-deletion loops and correct them [38] (Figure 2). It has been shown that mutation rates of MS are increased in MMR deficient cell lines compared to MMR proficient ones [39]. MMR deficiency could be attributed to mutations, epigenetic changes or post-translational modifications, as mentioned previously. MSI in HNPCC has been correlated to mutations within *MMR* genes [14], and suppressed expression of MMR proteins has been found in several types characterized as MSI-high cancers and cancer cell lines [40–42]. Suppressed expression of *MMR* could also be due to epigenetic changes, as many MSI-high sporadic cancers have been found to lack *hMLH1* expression because of methylation of *hMLH1* gene promoter [43,44]. Although mutations and silencing of *MMR* genes are usually responsible for high levels of MSI, low levels of MSI have been observed in some cancers with no known *MMR* mutations or promoter hypermethylation [14] thus, proving the existence of alternative mechanisms of DNA repair deactivation. MMR deficiency can be attained also without mutation or epigenetic change through a deregulation of expression of one of its subunits. MSH2 forms two different complexes with MSH3 and MSH6 with different activity. MSH3 and MSH6 compete with each other for binding with MSH2. Thus, overexpression of either of MSH3 or MSH6 can lead to decreased activity of the other’s complex with MSH2 [6]. Very recently, miR-155, a micro-RNA, which is over-expressed in MSI-high colorectal cancers, was found to down-regulate MMR protein levels and leads to MSI induction, suggesting that MMR levels could also be regulated in a post-transcriptional level [45]. In the subset of chronic tissue stress, ROS (reactive oxygen species) or RNS (reactive nitrogen species) may impair MMR function by post-translational modifications of MMR proteins [28].

Although the MMR system is responsible for correcting DNA strand loops typical for MSI, recent studies suggest that the different DNA repair systems do not act independently and that MSI may be attributable to alterations in DNA repair pathways distinct from MMR [46,47]. In addition, since all the types of DNA repair mechanisms use the same types of polymerases for final DNA synthesis [38], excess activity of one repair mechanism may result in reduction of the activity of the other. Hofseth *et al*. demonstrated in an elegant study that the chronic inflammation in ulcerative colitis leads to MSI through excessive activity of BER enzymes and, therefore, insufficient MMR activity [46]. It has been also shown that imbalanced expression of Polβ can be associated with MSI and chromosome instability [47,48]. The exact DNA repair defect behind the observed genomic instability found in allotransplanted patients needs to be elucidated. Interestingly, according to a recent study, patients with specific SNPs in BER genes exhibited higher risk for severe acute GvHD and chronic GvHD occurrence [49]. In addition, normal human CD34+ hematopoietic progenitors were found to lose MLH1 expression due to acquired promoter hypermethylation in the process of normal aging and exhibit MSI [50]. These data indicate that GvHD may develop in an already defective DNA repair cellular background.

### 3.3. Failure of DNA Damage Checkpoints

Besides the DNA repair proteins, cells are equipped with additional DNA protective mechanisms (ATM, p53 *etc*.), which detect unrepaired DNA and lead the cell to cycle arrest or apoptosis [7]. So, even in the case of repair mechanisms failure to correct DNA damage, cells which exhibit genomic instability (GI) will not further proliferate, and thus, they shouldn’t be detected among the rest of the normal cells, which proliferate normally. Inactivation of p53 and the failure of cellular demise have been suggested to play a mechanistic role in the occurrence of MSI in sporadic tumors and in chronically inflamed tissue [14,19,51–53]. It has been reported that cells with defective *MMR* genes fail to initiate cell cycle arrest in response to DNA damage. The molecular basis of this phenomenon is not completely known, however it has been shown that MMR deficient cells fail to phosphorylize p53 and p73 after DNA damage [38]. Furthermore, it is interesting that solid tumors post-transplant exhibit *p53* mutations [22,54]. The pattern of *p53* expression in GvHD-affected and genome instable epithelium in allotransplanted patients needs further evaluation.

### 3.4. Growth Advantage of GI Clones in the Allotransplanted Recipients

Another interesting hypothesis is whether cells with instable MS gain an immunologic survival advantage in contrast to normal cells, and thus, they can be detected after allo-HSCT. *In vitro* experiments have shown that cells that display MSI can escape T-cell mediated destruction through inhibition of HLA antigen expression and abnormal presentation of peptide fragments [55,56]. The inhibition of *HLA* expression can be caused by mutations in HLA transcriptional areas or by instability of microsatellites located near the *HLA* gene complex. After allogeneic HCT, the recipient’s epithelial cells stimulate through the HLA system the new immune system, which derives from the donor. This could lead to immune mediated epithelial cell destruction, which we recognize clinically as GvHD. Therefore, if genomic instable cells reduce expression of *HLA* genes, then they will acquire an immunologic survival advantage. Whilst the normal cells will undergo destruction by the donor-derived immune system, the MSI displaying cells will not be recognized by the donor’s T-cells. This hypothesis is surely challenging and should be further investigated.

## 4. Clinical Significance of GI in Allotransplanted Patients

MSI has been shown to be an indicator of genomic instability [9]. It is very likely that mutations occur not only in non-transcribed MS, but also in coding regions of the genome [57]. Mutations in coding genes might be responsible for the protean post-transplant GvHD-related clinical syndromes and phenotypes, such as scleroderma, Sjögren syndrome, musculoskeletal or pulmonary disease and many others. In addition to serving as an indicator for genomic instability, mutations of microsatellites may directly contribute to evolution of post-transplant diseases. Although microsatellites are thought to be part of the “junk” DNA dispersed in non-coding regions throughout the genome, there is now growing evidence that non-coding DNA distribution is not random. As shown by the accumulating data of the ENCODE project, the previously thought to be “useless” DNA in fact contains functional elements rich in protein and RNA binding sites and brings them to the ideal conformation to regulate the function and expression of protein-coding genes [58]. Therefore, it seems that a potential change of a microsatellite length could end up in possible relevant changes of the cellular phenotype. In addition, it is already known that there are microsatellite sequences within genes, which play a regulatory role in gene expression [11]. *TGFRII*, *IGFIIR* and *BAX* genes are paradigms of transcribed genes, which contain microsatellites within their coding regions [14,57]. Changes in the lengths of these repetitive sequences, when found within coding regions of specific genes, have been shown to result in gene inactivation or modification of function of the gene and cause disease in humans such as Huntington’s disease and myotonic dystrophy [57].

The detection of MSI in clinical samples has been mainly associated with cancer [14] or cancer risk in chronic inflammatory diseases [20]. In hereditary nonpolyposis colorectal carcinoma, the MMR deficiency plays an aetiological role in the development of MSI and carcinogenesis [14]. Microsatellite alterations are also common in various sporadic cancers, such squamous cancers [14]. It is interesting that squamous cell carcinomas are the most common secondary tumors after allogeneic HSCT [59] and are associated with chronic alloantigeneic stimulation through GvHD. Statistical analysis of clinical data in a recent study by our group showed a significant correlation of MSI detection in the epithelium of allotransplanted patients and the development of secondary squamous cell neoplasia. This observation enhances the possibility of a pathogenetic role of MSI in secondary malignancy development. Deeg *et al.* found in eight post-transplant tumors a p53 expression indicative for mutations of the *p53* gene [54]. Themeli *et al*. [22] found *p53* mutations in two out of four post-transplant tumors, but no *p53* mutations in six samples where MSI was detected. These findings indicate that MSI occurrence is an early phenomenon after allo-HSCT. Therefore, the hypothesis that MSI may characterize a precancerous state of the epithelial cell seems logical. The mutator phenotype may further contribute to the development of new mutations in coding regions with important function, such as *p53* gene, and promote carcinogenesis. Genome-wide analyses in allografted recipients may indeed identify specific genomic alterations, which might be responsible for or used as molecular biomarkers of post-transplant diseases, including secondary cancer.

## 5. Conclusions

**Figure 3:**
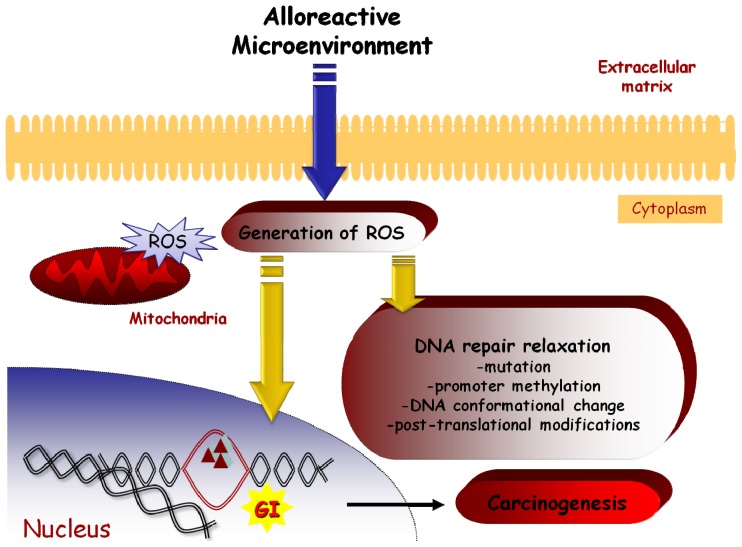
Schematic representation of a possible GI induction mechanism in epithelial tissues by an alloreactive microenvironment.

Taken together, it seems that in the GvHD tissue environment cytokines, ROS and NO induce genomic alterations in epithelium by acting through several different, but communicating, pathways. The oxidative stress damages the DNA, causing mutations, such as microsatellites length alterations. The induction of mutations is facilitated by a relaxation of DNA repair system mediated by NO or ROS. The cells may survive due to a possible inactivation of the DNA-checkpoint apoptotic mechanisms. Furthermore, the donor-derived immune system in an allogeneic setting may attack the recipient-derived normal epithelial cells, yet spare cells with an MSI phenotype, providing in this way a selective growth advantage for the cells with novel repeat lengths (Figure 3). Elucidating the ultimate mechanisms underlying the genomic instability following alloantigeneic reactions in the chimeric organism is a major challenge. Findings may provide more information about GvHD pathology and pathogenesis of post transplantation clinical outcomes, such as secondary malignancies and GvHD related syndromes. Focusing on the pathways through which the alloantigeneic reaction causes genomic instability may bring up novel therapeutic targets for the protection of the epithelium during GvHD and the prevention of malignant transformation.
